# Ataxia-associated DNA repair genes protect the *Drosophila* mushroom body and locomotor function against glutamate signaling-associated damage

**DOI:** 10.3389/fncir.2023.1148947

**Published:** 2023-07-05

**Authors:** Ilse Eidhof, Alina Krebbers, Bart van de Warrenburg, Annette Schenck

**Affiliations:** ^1^Department of Human Genetics, Donders Institute for Brain, Cognition, and Behaviour, Radboud University Medical Center, Nijmegen, Netherlands; ^2^Department of Neurology, Donders Institute for Brain, Cognition, and Behaviour, Radboud University Medical Center, Nijmegen, Netherlands

**Keywords:** autosomal recessive cerebellar ataxia, *Drosophila*, locomotor behavior, DNA repair, glutamate signaling, mushroom body

## Abstract

The precise control of motor movements is of fundamental importance to all behaviors in the animal kingdom. Efficient motor behavior depends on dedicated neuronal circuits – such as those in the cerebellum – that are controlled by extensive genetic programs. Autosomal recessive cerebellar ataxias (ARCAs) provide a valuable entry point into how interactions between genetic programs maintain cerebellar motor circuits. We previously identified a striking enrichment of DNA repair genes in ARCAs. How dysfunction of ARCA-associated DNA repair genes leads to preferential cerebellar dysfunction and impaired motor function is however unknown. The expression of ARCA DNA repair genes is not specific to the cerebellum. Only a limited number of animal models for DNA repair ARCAs exist, and, even for these, the interconnection between DNA repair defects, cerebellar circuit dysfunction, and motor behavior is barely established. We used *Drosophila melanogaster* to characterize the function of ARCA-associated DNA repair genes in the mushroom body (MB), a structure in the *Drosophila* central brain that shares structural features with the cerebellum. Here, we demonstrate that the MB is required for efficient startle-induced and spontaneous motor behaviors. Inhibition of synaptic transmission and loss-of-function of ARCA-associated DNA repair genes in the MB affected motor behavior in several assays. These motor deficits correlated with increased levels of MB DNA damage, MB Kenyon cell apoptosis and/or alterations in MB morphology. We further show that expression of genes involved in glutamate signaling pathways are highly, specifically, and persistently elevated in the postnatal human cerebellum. Manipulation of glutamate signaling in the MB induced motor defects, Kenyon cell DNA damage and apoptosis. Importantly, pharmacological reduction of glutamate signaling in the ARCA DNA repair models rescued the identified motor deficits, suggesting a role for aberrant glutamate signaling in ARCA-DNA repair disorders. In conclusion, our data highlight the importance of ARCA-associated DNA repair genes and glutamate signaling pathways to the cerebellum, the *Drosophila* MB and motor behavior. We propose that glutamate signaling may confer preferential cerebellar vulnerability in ARCA-associated DNA repair disorders. Targeting glutamate signaling could provide an exciting therapeutic entry point in this large group of so far untreatable disorders.

## 1. Introduction

The precise control of motor movements is of fundamental importance to all behaviors in the animal kingdom and its efficiency depends on dedicated neuronal circuits. The development, function and maintenance of motor circuits are controlled by extensive genetic programs. When affected by mutations, these can lead to motor circuit dysfunction and affect output behavior. Whether the genetic programs that control motor behavior are evolutionarily conserved, and how their interactions maintain specific motor circuits is incompletely understood.

Autosomal recessive cerebellar ataxias (ARCAs) represent a valuable disease-related entry point into the genetics controlling motor circuit function. ARCAs are a clinically and genetically heterogenous group of disorders, characterized by impaired motor coordination of all body effectors due to dysfunction and eventual degeneration of the cerebellar circuitry (Jayadev and Bird, 2013). There is a wide range of onset age, but these diseases often manifest early (<age of 20) and primarily as progressive disturbances of gait, balance, limb coordination, oculomotor control, and speech that gradually lead to a loss of independence (Jayadev and Bird, 2013). Often, there is involvement of other central and peripheral nervous system structures and even other organ systems. There is still no treatment for the large majority of ARCAs to counteract the devastating consequences of these disorders in daily life.

We previously investigated whether ARCA-associated genes operate in common biological pathways and found a striking enrichment of genes involved in DNA repair (Eidhof et al., 2018b,c). Functional DNA repair may thus be an evolutionary conserved biological process that is particularly relevant for the preservation of cerebellar motor circuits. Expression of these genes in the cerebellum however, is not significantly different from expression in other parts in the brain (Eidhof et al., 2018b,c). How dysfunction of ARCA-associated DNA repair genes preferentially affect the cerebellar circuitry and subsequently motor behavior is therefore unclear. Only a few animal models for DNA repair ARCAs have been generated, and even for these, the interconnection between defects in DNA repair, their cerebellar circuit dysfunction, and motor behavior is barely established (Katyal et al., 2007; Hawkins et al., 2009; Tal et al., 2018).

To examine the relevance of ARCA-associated DNA repair pathways in motor function and cerebellar-like circuits, we turned to the fruit fly *Drosophila melanogaster.* A number of parallels have been drawn between the vertebrate cerebellum and the *Drosophila* mushroom body (MB), both on the structural and functional level (Farris, 2011). Both are involved in a variety of experience-dependent adaptive behaviors, and are wired accordingly, showing a number of similar properties (Farris, 2011; Riemensperger et al., 2013; Bushey et al., 2015; Sun et al., 2018). The enormous number of cerebellar granule cells (GCs) receive sensory input from only a small set of mossy fibers (MFs), in a ratio 30:1. This sparse expansion ratio is very similar to the sparse synaptic sensory input that Kenyon Cells constituting the MB receive. In turn, single Cerebellar Purkinje output and single MB output neurons receive synaptic input from many Granule and Kenyon cells, respectively. The sparse representation of sensory information in the cerebellum and MB has been theorized to optimize the discrimination of stimuli and to enhance perceptual processing power, to control efficient motor coordination. Currently, it is unclear which types of motor behaviors the MB controls. It is also unknown whether ARCA-associated DNA repair genes operate in the MB to regulate motor behavior.

Here, we propose that the MB is required for efficient startle-induced and spontaneous motor behaviors. Inhibition of synaptic activity and loss-of-function of ARCA-associated DNA repair genes in the MB affected motor behavior in several assays. The identified motor deficits correlated with increased levels of MB DNA damage, MB neuron apoptosis and/or alterations in MB morphology at later stages of development. We further propose that glutamate signaling may confer preferential cerebellar vulnerability in ARCA-DNA repair disorders. We show that expression of glutamate signaling pathways are highly, specifically, and persistently elevated in the postnatal human cerebellum and that manipulation of glutamate signaling in the MB induces motor defects, MB neuron DNA damage and apoptosis. Importantly, reduction of glutamate signaling in the ARCA DNA repair models rescued the identified motor deficits. Our findings suggest that glutamate excitotoxity instigates cerebellar vulnerability in ARCA-DNA disorders.

## 2. Materials and methods

### 2.1. Fly stocks and breeding

Fly stocks were maintained at RT on standard *Drosophila* medium (cornmeal, sugar and yeast). Crosses were raised at 28°C, 60% humidity, 12:12 h light-dark cycle. Inhibition of synaptic transmission in (subsets of) mushroom body neurons was induced by crossing virgins of w*; P{UAS-TeTxLC.tnt}G2; (from now on referred to as UAS-TeTx) or the control line w*; P{UAS-TeTxLC.(-)V}A2; (from now on referred to as UAS-impTeTx) with males of w*;R13F02-Gal4 (Bloomington *Drosophila* Stock Center (BDSC) stock #48571), w*,UAS-Dicer-2;247-Gal4 (kindly provided by Krystyna Keleman) or Split-Gal4 lines MB010B, MB008B, MB185B, MB477B, MB594B, MB418B, MB463B, MB009B, MB419B, or MB607B [FlyLight collection, kindly provided by Janelia (HHMI)] (Aso et al., 2014). UAS-TeTx and UAS-impTeTx fly strains (BDSC stocks #28838 and #28840) were isogenized for at least 6 generations with the Vienna *Drosophila* Resource Centre (VDRC) 60,000 background control. For all experiments, induced UAS-TeTx flies were compared to their induced UAS-impTeTx control flies in the same genetic background (progeny of promotor lines crossed with UAS-impTeTx).

Mushroom body-specific knockdown was achieved by crossing virgin females from the driver line *w;UAS-Dicer-2;247-Gal4* to UAS-RNAi males obtained from VDRC. In all experiments, knockdown flies were compared to their appropriate genetic background controls (progeny of the promoter line crossed with the genetic background of the VDRC GD (VDRC #60000) or KK (VDRC #60100) RNAi library). All RNAi constructs had an s19 value of 1.00, and zero predicted off-targets. The genomic integration site of all VDRC KK RNAi library lines were characterized by PCR as previously described and found to be at landing site 30B3, not prone to reported potential dominant phenotypes unrelated to RNAi (Green et al., 2014; Vissers et al., 2016). Because the VDRC GD control background crossed with the 247-Gal4 driver performed rather poorly in our behavioral assays, we avoided using RNAi lines of the VDRC GD library where KK lines were available [(for mre11 and Gad1 only GD lines were available)]. The following RNAi strains were used in this study: tefu-RNAi (VDRC #108074), mre11-RNAi (VDRC #30474), gkt-RNAi (VDRC #109757), Aptx-RNAi (VDRC #108346), Pnkp-RNAi (VDRC #108251), mGluR-RNAi (VDRC #103736), VGlut-RNAi (VDRC #104324), and Gad1-RNAi (VDRC #32344).

### 2.2. Island assay

The island assay was used to evaluate the locomotor behavior of 4-days-old male *Drosophila* models of ARCA DNA repair genes as described, during Zeitgeber Time 3–8 (Eidhof et al., 2017, 2018a). In brief, 4 days old male adult flies were thrown onto an elevated platform surrounded by soap water and their flight escape response was video recorded. The number of flies remaining on the platform was quantified every 0.1 s using an in-house developed automatic script. All behavioral experiments were performed at room temperature under standard light conditions. For each genotype, three biological replicates were investigated.

### 2.3. Locomotion assay

The locomotion assay was performed as described (Eidhof et al., 2018a), with the exception that tracking was carried out at room temperature, 3 days after collection during Zeitgeber Time 3–8. Locomotion of 4-days-old male flies was tracked using the semi-automatic machine-vision program Ctrax (Version 0.5.18) (Branson et al., 2009). Distances within the videos were calibrated based on the diameter of the arena. The Ctrax output files were further analyzed in Matlab, to calculate total distance and speed while moving. For each genotype, a minimum of 28 flies were analyzed per biological replicate. Three biological replicates were analyzed.

### 2.4. Activity monitoring with DAM system

Individual, 4-days-old male flies were placed without anesthetization in 65 mm × 5 mm glass tubes (Trikinetics) containing standard food. Activity counts were recorded the next day with the *Drosophila* Activity Monitor (DAM) system (Trikinetics, model DAM2) for a duration of 4 days under 12:12 h light: dark conditions. Activity counts were binned in 30 min intervals with DAMFileScan software (Trikinetics). For each genotype, a minimum of 14 flies were analyzed per experiment. Experiments were repeated three times.

### 2.5. Immunostaining and immunofluorescence microscopy

To detect cell death, differences in MB morphology and DNA damage, brains of 20-days-old male flies were dissected and fixed for 30 min in 3.7% PFA, rinsed twice in 1× PBS and blocked for 2 h in PBS-Triton X-100 (0.3%, PBS-T) containing 5% NGS at room temperature. Brains were incubated with the primary antibodies anti-FasII (DSHB, 1:5) or anti-dac (mAbdac1-1, DSHB, 1:125) in combination with anti-Asp175 (Cell signaling, 1:200) or anti-γH2AvD pS137 (Rockland, 1:200) for 2 d at 4°C. Brains were washed 5 times for 10 min in PBS-T and incubated for 2 h with secondary antibodies Alexa-568 red mouse (Life Technologies, 1:500) and Alexa-488 green rabbit (Invitrogen, 1:500) at room temperature. Brains were mounted in ProLong Gold Antifade reagent (ThermoFisher Scientific). At least 20 images per genotype were acquired with a Zeiss Axio Imager fluorescence microscope (63 × magnification for anti-dac and anti-Asp175, and 40× magnification for anti-FasII immunostaining with an apotome, and a distance of 1 μm between imaged fields. All images were further analyzed with Fiji.

### 2.6. BrainSpan developmental transcriptome analysis

The publicly available developmental transcriptome RNA sequencing (RNA seq) data from the Human BrainSpan atlas were used to identify processes highly and specifically elevated in the cerebellum. BrainSpan provides RNA seq count data represented as reads per kilobase per million mapped reads (RPKM) of 11 neocortical human brain regions and five targeted non-neocortical brain regions. Details on samples, sequencing protocols and RNA expression acquisition can be found at the brainspan website.^1^ First, low expressed genes, with an average expression <0.05 RPKM over brain developmental stages and regions, were filtered out. Data were then binned into five stages, across important developmental periods of the postnatal human brain (Eidhof et al., 2018b). EdgeR (version 3.16.5) and Limma (version 3.30.7), provided by the online service Bioconductor, were used to identify differentially expressed genes in pairwise comparisons between the 16 brain regions over the developmental stages. Genes were considered to be highly elevated between two brain regions if their LogFC threshold >10 and adjusted *p*-value passed the <0.05 threshold (after Benjamini-Hochberg correction for the number of elevated genes) (Supplementary Table 1).

### 2.7. Gene ontology analysis

The webtool G-profiler (Reimand et al., 2016) (rev 1,760, build 10/2018) was used to perform Gene-Ontology (GO) analysis. As background, the filtered gene list (all genes with an average RPKM >0.05 over all developmental stages) was used. Only GO terms (Biological Process, Molecular Function and Cellular Component) that were significantly enriched after correction for multiple testing (Bonferroni test, *p* < 0.05) were considered.

### 2.8. Enrichment analysis

Enrichment scores for GO-terms were calculated as followed: (*a*/*b*)/[(*c–a*)/(*d–b*)], where *a* is the number of elevated genes associated with that GO-term, *b* is the total number of elevated genes, *c* is the total number of genes associated with that GO-term remaining after filtering out low expressed genes from BrainSpan data and *d* is the total number of genes remaining after filtering out low expressed genes from BrainSpan data (16,956 genes).

### 2.9. Analysis of expression data of isolated nuclei of specific MB neuronal subtypes

For analysis of gene-expression in isolated nuclei of specific MB neuronal subtypes, expression data was downloaded from Shih et al. (2019). Per gene of interest, expression values in different MB neuronal subtypes were normalized against the average Counts Per Million (CPM) value of that specific gene over all MB neuronal subtypes.

### 2.10. Riluzole compound testing

Freshly eclosed male flies were collected and transferred to either vials with standard food containing 5 mM riluzole (prior diluted in DMSO, to an end concentration of 1%) or vials containing standard food supplemented with 1% DMSO (mock food). After 4 days on these regimes, flies were subjected to the open-field arena assay and their walking abilities were evaluated as described previously. For each experimental condition, a minimum of 28 flies were analyzed per experiment. Experiments were repeated three times.

### 2.11. Statistical analysis

GraphPad Prism software and the Kruskal–Wallis test with Dunn’s multiple comparisons test was used to calculate significant differences between more than two conditions. Non-parametric *t*-test was used to calculate differences between two conditions; *p*-values < 0.05 were considered to be significant.

## 3. Results

### 3.1. The *Drosophila* mushroom body is involved in the regulation of motor behavior

**FIGURE 1:**
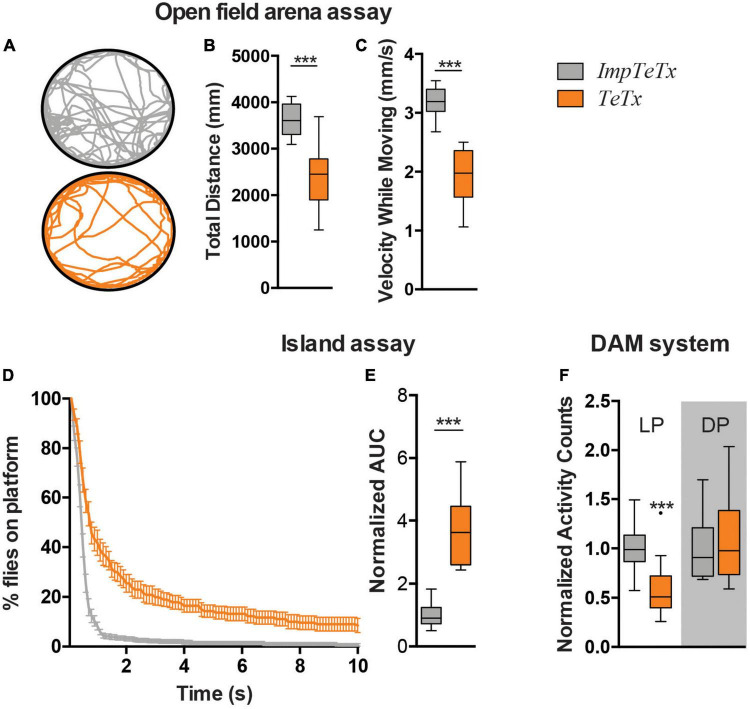
Inhibition of synaptic transmission in the *Drosophila* mushroom body neurons affects motor behaviors. **(A)** Representative trajectory of an individual fly in the open field arena. **(B)** Total distance moved in mm in the assay (7 min). **(C)** Speed while moving (mm/s). **(D)** Island assay: % of flies that remained on the platform over time. **(E)** Island assay quantification: Area Under the Curve (AUC) of graphs shown in panel **(D)**. Data is normalized against the control (TeTx/ImpTeTx). **(F)** Activity counts, measured with the DAM system. Light Period (LP) and Dark Period (DP). Data is normalized against the control (TeTx/ImpTeTx) in the respective period. ***p < 0.001. Age of flies: 4 day. Genotypes are ImpTeTx (in gray): ;*UAS-impTeTx/* + *;R13F02-Gal4/* + (genetic background control flies expressing impaired Tetanus toxin) and TeTx (in orange): ;*UAS-TeTx/* + *;R13F02-Gal4/* + (synaptic transmission inhibited by expression of Tetanus toxin).

Locomotor behavior is regulated by the MB (Sun et al., 2018). To determine the relevance of the *Drosophila* MB for startle-induced flight behavior in the island assay, we expressed tetanus toxin (TeTx) using MB drivers 247-Gal4 and R13F02-Gal4 to block synaptic transmission (Sweeney et al., 1995). The 247-Gal4 driver is strongly, and with relatively high specificity, expressed in α-, β- and γ-MB neurons, but is not expressed in α’- and β’-MB neurons. The R13F02-Gal4 driver is an overall weaker driver than 247-Gal4, but it targets all MB neurons (Jenett et al., 2012; Pech et al., 2013; Aso et al., 2014). *UAS-Dcr-2/UAS-TeTx;247-Gal4* flies were lethal; no adult progeny was obtained that could be tested in the assay. In contrast, TeTx expression by the R13F02-Gal4 driver gave rise to adults. Measured in an open field arena, ;*UAS-TeTx;R13F02-Gal4* flies moved significantly less upon startle-induced stimuli (*p* < 0.001) and with slower speed (*p* < 0.001) (Figures 1A–C). In the island assay, these flies showed as well a significantly reduced startle-induced motor performance, compared to their isogenic controls that express impaired tetanus toxin (impTeTx) (Figure 1D; Sweeney et al., 1995). Quantification of these defects based on calculating the area under the curve (AUC) of the percentage of flies remaining on the platform, revealed a significant fold-change of 3.5, *p* < 0.001 (Figure 1E).

We next addressed whether inhibition of synaptic transmission in the MBs also affects other aspects of locomotion such as spontaneous locomotor activity in the *Drosophila* Activity Monitoring (DAM) system. ;*UAS-TeTx;R13F02-Gal4* flies passed the infrared beam significantly less often during the respective day period compared to impTeTx-expressing controls (*p* < 0.001), but showed no difference in the night period (Figure 1F). In agreement with other studies, these data implicate the *Drosophila* MBs in three forms of locomotor behavior, including both spontaneous and startle-induced locomotor behaviors.

We continued determining whether the effect of inhibiting synaptic transmission in the MB on motor behavior could be potentially caused by changes in MB morphology and increased levels of Kenyon cell apoptosis. Even when assessed after 20 days of TeTx induction, there were no visible differences in the overall morphology of the MB α-, β- and γ-lobes. Detailed quantification of their length and width revealed a small increase in the width of MB γ-lobes (*p* < 0.001) as the only altered parameter (Supplementary Figure 1A). To evaluate whether continuous inhibition of synaptic transmission in the MB causes increased levels of Kenyon cell apoptosis, we performed co-immunolabeling using antibodies against Caspase-3 cleaved at Asparagine 175, a marker of apoptosis, and against dac visualizing Kenyon cell nuclei. No increase in Asp175-positive Kenyon cells was observed after 20 days of TeTx induction (Supplementary Figure 1B). In conclusion, inhibition of synaptic transmission in the MB does neither affect overall MB morphology nor induces Kenyon cell apoptosis under the given experimental conditions. It is thus conceivable that reduced synaptic transmission itself is the acute cause of the behavioral defects.

Startle-induced and spontaneous motor behaviors depend on different input pathways that might affect different neuronal subsets in the MB (Riemensperger et al., 2013; Sun et al., 2018). We therefore questioned whether the affected types of motor behavior can be localized to specific neuronal subsets within the MB. To test this, we employed a set of ten Split-Gal4 drivers from the Flylight collection, which are highly and specifically expressed in the different MB lobes and together cover all neuronal subsets in the MB (Aso et al., 2014). These were used to inhibit synaptic transmission in a spatially restricted manner, and startle induced and spontaneous motor behavior was assessed in the island assay and DAM system, respectively. Inhibition of synaptic transmission with each of the 10 tested MB Split-Gal4 lines faithfully reproduced the defects induced by the MB-wide driver in island assay behavior, suggesting that all tested subsets of MB neurons affect startle-induced motor behavior (Supplementary Figure 2B). In contrast, we were unable to recapitulate the reduced activity counts during light periods upon inhibition of synaptic transmission with any of the ten MB Split-Gal4 lines in the DAM system. Instead, inhibition of synaptic transmission with nine of the ten lines resulted in a significant increase in activity counts during the dark period (Supplementary Figure 2C). Combined, these data suggest that an efficient startle-induced locomotor behavior in the island assay critically depends on proper assembly and/or function of all MB components. In contrast, spontaneous motor behavior in the DAM system was exclusively affected when blocking synaptic transmission using the R13F02 driver, spanning all MB neurons.

### 3.2. Loss of function of ARCA DNA repair machinery in the *Drosophila* mushroom body affects motor behaviors

**FIGURE 2:**
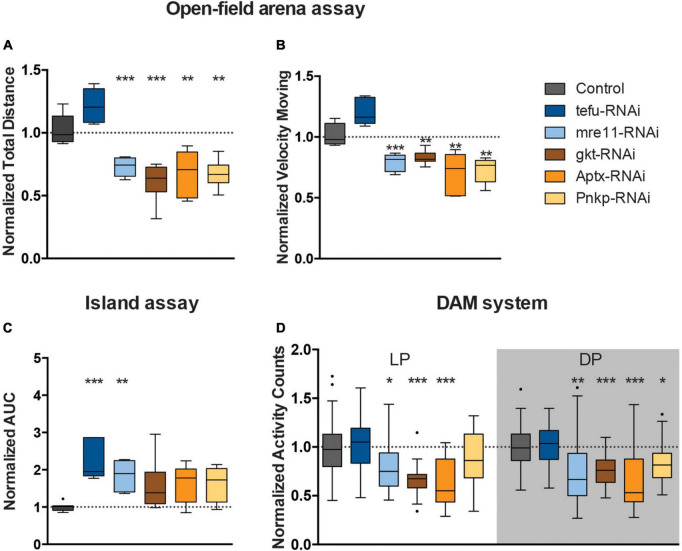
Loss of ARCA-associated DNA repair gene expression affects motor behaviors. **(A)** Total distance moved and **(B)** speed while moving in open field arena. Data is normalized against appropriate genetic background control (RNAi/Control). **(C)** Area Under the Curve for % of flies that remained on the platform during 10 s of island assay performance. Data is normalized against the appropriate control condition (RNAi/Control). **(D)** Displays normalized activity counts as measured with the DAM system, data normalized against control condition for Light Period (LP) and Dark Period (DP) (RNAi/Control) in the respective period. ***p < 0.001, **p < 0.01, and *p < 0.05. Age of flies: 4 days old. For information on genotypes, and n see section “2. Materials and methods”.

We next asked whether DNA repair genes that are mutated in ARCAs serve a conserved role in the control of efficient motor behavior, and whether this function resides in the MB. Table 1 describes the ARCA-associated DNA repair genes that we included in this study, their function in DNA repair, the associated human disorder, and their conservation in *Drosophila*. We attempted to model loss-of-function of ARCA-associated DNA repair genes in the MB by inducing RNA interference (RNAi) with the strong MB 247-Gal4 driver to target these genes and assessed spontaneous and startle-induced motor behavior. The 247-Gal4 driver contains an extra copy of UAS-Dicer-2, enhancing RNAi knockdown efficiency (Dietzl et al., 2007). Loss-of-function of all five ARCA-associated DNA repair genes in the MB induced phenotypes in startle-induced motor behavior, spontaneous motor behavior, or both (Figure 2).

**TABLE 1:** Summary of ARCA-associated DNA repair genes included in this study.

Disorder	Associated gene	Gene function	Orthology human:fly	Flygene
Ataxia-telangiectasia (AT)	*ATM*	Cell cycle checkpoint kinase that phosphorylates downstream effectors in DNA repair such as H2AX (H2Av in *Drosophila*).	1:1	*tefu*
Ataxia-telangiectasia-like disorder 1 (ATLD1)	*MRE11a*	Has 3′ to 5′ exonuclease activity, part of Rad50/MRE11/P95 complex [involved in DSB (NHEJ and HR)].	1:1	*mre11*
Spinocerebellar Ataxia, autosomal recessive with axonal neuropathy (SCAN1)	*TDP1*	Repairs topoisomerase I induced DNA damage by catalyzing the hydrolysis of phosphodiester bond between tyrosine residue and the DNA 3′ phosphate, and is involved in SSBR.	1:1	*gkt*
Ataxia-oculomotor apraxia (AOA1)	*APTX*	Catalyzes the nucleophilic release of adenylate groups linked to 5′-phosphate termini at single-strand breaks.	1:1	*Aptx (CG5316)*
Ataxia-oculomotor apraxia (AOA4)	*PNKP*	Catalyzes 5′-phosphorylation of nucleic acids and has 3′-phosphotase activity (involved in NHEJ and SSBR)	1:1	*Pnkp (CG9601)*

Of note, only high-confidence (>1 described family) ARCA-associated DNA repair genes, with a direct function in DNA damage signaling and repair were included.

Mushroom body knockdown of tefu, the ortholog of *ATM*, significantly decreased the ability of flies to leave the platform in the island assay. The AUC of the % flies remaining on the platform significantly increased to more than 2.5 fold (*p* < 0.001) compared to their genetic background controls (Figure 2C), suggesting a role for tefu-mediated DNA repair in startle-induced motor behavior in the island assay. Other motor behaviors, including startle and non-startle induced walking behavior, were not significantly affected upon tefu MB knockdown (Figures 2A, B, D).

Knockdown of mre11, the ortholog of *MRE11a*, affected both startle-induced and spontaneous motor behavior (Figures 2A–D). Analysis of the flies startle-induced walking behavior in the open field arena demonstrated that they walked a significantly shorter distance in a given time interval (30% reduction, *p* < 0.001) compared to the controls, and, when moving, also showed reduced velocity (∼20% reduction, *p* < 0.001) (Figures 2A, B). In the island assay, the AUC of the % flies remaining on the platform significantly increased to 1.8 fold (*p* < 0.01) compared to their controls (Figure 2C). In addition, mre11-RNAi flies passed the infrared beam significantly less frequently (>20% reduction) during the respective day (*p* < 0.05) and night period (*p* < 0.01) in the DAM system than the controls (Figure 2D).

Knockdown of gkt, the ortholog of *TDP1*, significantly affected walking behavior in startle-induced and spontaneous conditions (Figures 2A, B, D). In the open field arena, gkt-RNAi flies moved less (40% reduction, *p* < 0.001), and with slower speed (20% reduction, *p* < 0.01) (Figures 2A, B). Further, Gkt-RNAi flies passed the infrared beam in the DAM system 40% less frequently during light period (*p* < 0.001) and 20% less frequently during the dark period (*p* < 0.001) compared to control flies (Figure 2D).

Knockdown of Aptx, the ortholog of *APTX*, significantly affected startle and spontaneous induced walking behavior (Figures 2A, B, D). Examination of walking behavior of Aptx-RNAi flies in the open field arena upon startle induced stimuli, demonstrated that these flies walked a significantly shorter distance in a given time interval (33% reduction, *p* < 0.01) compared to the controls, and, when moving, also showed reduced velocity (30% reduction, *p* < 0.01) (Figures 2A, B). In the DAM system, Aptx-RNAi flies showed a 40% reduction in activity counts in both light (*p* < 0.001) and dark (*p* < 0.001) periods compared to the control (Figure 2D).

Knockdown of Pnkp, the ortholog of *PNKP*, resulted in significant less motion (28% reduction, *p* < 0.05), and reduced speed (28% reduction, *p* < 0.01) in the open-field arena (Figures 2A, B). Pnkp-RNAi flies also displayed affected activity counts (20% reduction, *p* < 0.05) during the dark period in the DAM system, indicating that Pnkp knockdown in the MB affects startle and spontaneous walking behavior (Figure 2D).

In summary, MB 247-Gal4-driven knockdown of ARCA-associated DNA repair genes induces defects in motor behavior. The extent of this essential role and the motor phenotypes observed upon loss-of-function of these genes were, to a certain degree, heterogeneous for the five genes investigated.

### 3.3. Loss of ARCA DNA repair genes in the mushroom body affect MB integrity

**FIGURE 3:**
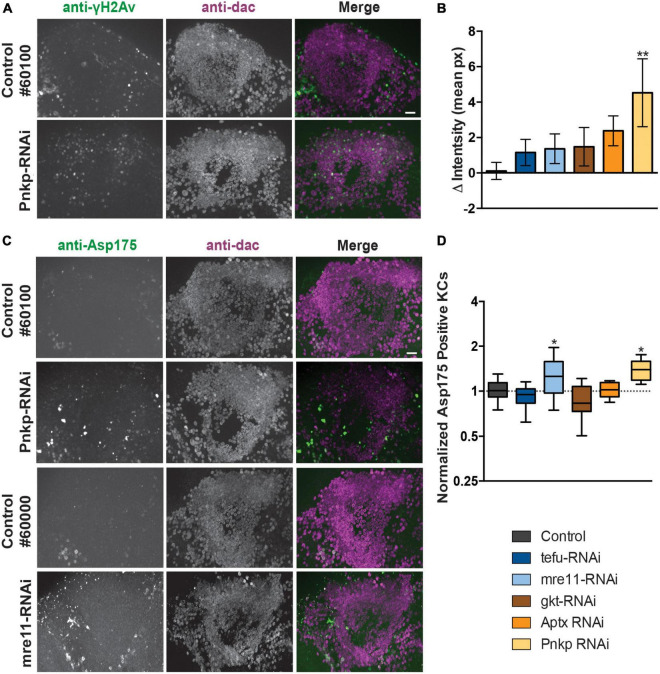
Loss of ARCA-associated DNA repair genes in the mushroom body increases DNA damage and apoptosis. **(A)** Anti-γH2Av and anti-dac co-immunolabeling of brains of indicated genotypes. Scale bar: 10 μm. **(B)** Quantification of DNA damage staining from microscopy images as shown in panel **(A)**: mean γH2Av intensity in dac-positive Kenyon cell area – γH2Av intensity outside Kenyon cell area of the indicated genotypes. **(C)** Anti-Asp175 and anti-dac co-immunolabeling of brains of indicated genotypes. Scale bar: 10 μm. A + C. Superimposed optical sections are shown. **(D)** Min-to-max boxplots displaying average number of dac-positive Kenyon cells that were positive for the cleaved-caspase-3 Asp175 marker for indicated genotypes. Data is normalized against the appropriate genetic background control condition. *p < 0.05, **p < 0.01, age of flies: 20 day. For information on genotypes, see section “2. Materials and methods”.

To determine if knockdown of ARCA DNA repair genes leads to increased Kenyon cell DNA damage, we performed co-immunolabeling with an antibody against phosphorylated H2Av (γH2Av), a histone modification serving as biomarker for double stranded DNA breaks (DSBs) and sites of DNA repair foci and anti-dac to visualize Kenyon cells. Preparations were imaged and, DNA DSB damage levels were quantified by subtracting the mean γH2Av pixel intensity within the Kenyon cell area from the mean intensity (in pixels) of the rest of the brain. Following this procedure, a slight increase in γH2Av intensity in the Kenyon cell area was observed upon knockdown of most ARCA-associated DNA repair genes, but was only significant after correction for multiple testing upon knockdown of Pnkp (*p* < 0.01) (Figures 3A, B). This suggests that knockdown of ARCA-associated DNA repair genes in the MB can lead to an increase in double-stranded DNA breaks, as assessed by γH2Av. In addition, single-stranded DNA damage may occur.

To determine whether increased levels of DNA damage in Kenyon cells correlated with increased levels of apoptosis, Kenyon cells were co-labeled with a marker of active apoptosis (antibody against cleaved Caspase-3 at Asparagine 175) and anti-dac. Knockdown of both mre11 and Pnkp led to a significant increase (*p* < 0.05) of apoptosis as revealed by Asp175 positive Kenyon cells, while no increase was detected in the other three models (Figures 3C, D).

We continued determining whether the effect of ARCA-associated DNA repair genes in the MB on motor behavior coincided with changes in MB morphology. The overall architecture of the MB seemed intact upon knockdown of each of the five ARCA-associated DNA repair genes. However, detailed quantification of the length and width of α-, β- and γ-lobes revealed a small increase in the length of MB β-lobes (*p* < 0.05) and decreased width of MB γ-lobes (*p* < 0.01) upon knockdown of mre11 compared to the genetic control (Supplementary Figures 3A–C). These results are well compatible with a certain level of loss of MB neuron’s integrity upon degeneration.

In conclusion, knockdown of ARCA-associated DNA repair genes affects the integrity of the MB. In agreement with their function in DNA repair, the exact observed phenotypes were, to a certain degree, heterogeneous for the five genes investigated. We note that some of the differences, most importantly the lack of yH2AV despite apoptosis in mre11 knockdown MB, is however, readily explained by the gene’s function. We refer to our discussion for a more thorough explanation.

### 3.4. Glutamate signaling pathways are highly, specifically, and persistently elevated in the postnatal cerebellum

**FIGURE 4:**
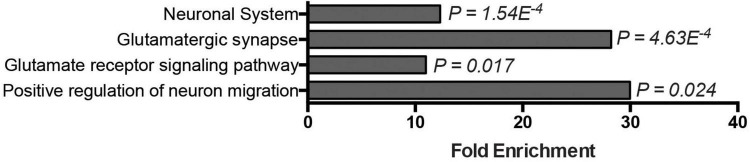
Glutamate signaling–related genes are highly, specifically, and persistently elevated in the postnatal cerebellum. Bar graph displays GO-terms significantly enriched among genes specifically expressed in the postnatal cerebellum (>10-Logfold enrichment throughout postnatal stages, *p-adj.* < 0.05).

The basis for the preferential regional vulnerability of cerebellar motor circuits in DNA repair-associated ARCAs is poorly understood. The expression of most ARCA-associated DNA repair genes is neither specific to nor particularly enriched in the cerebellum (Eidhof et al., 2018b), suggesting that other properties make the cerebellum more vulnerable to defects in DNA repair pathways. We therefore asked which genes and pathways are in general highly and specifically upregulated in the cerebellum that could potentially account for this vulnerability. To address this, we turned to the publicly available BrainSpan Transcriptional Atlas of the Developing Human Brain, as a resource. After exclusion of low expressed genes (see section “2. Materials and methods”), a transcription matrix of 16,956 genes representing the 16 brain regions was left that was binned into five different postnatal developmental stages. We then calculated for each of the 16,956 genes per developmental period, whether it was differentially expressed in the cerebellum compared pairwise to any of the other 15 brain regions. From the resulting matrix, genes were extracted that showed at least a 100-fold higher expression in the cerebellum (adj. *p* < 0.05) compared to all of the other 15 brain regions, and did so over all five developmental stages. These criteria led to the identification of 131 genes (Supplementary Table 1). We next asked which functional biological modules were underlying this group of genes by performing GO term analysis. Genes that were highly (>100 fold), specifically (in cerebellum versus all other 15 brain regions) and persistently (throughout postnatal developmental stages) elevated in the postnatal cerebellum were significantly enriched in neuronal and glutamate-related processes: neuronal system (*p* = 1.54E^–4^), glutamatergic synapse (*p* = 4.63E^–4^), glutamate receptor signaling pathway (*p* = 0.017), and positive regulation of neuron migration (*p* = 0.024) (Figure 4). To determine how unique these GO-terms were for upregulated genes in the cerebellum, we repeated the same stringent analysis for all other 15 brain regions. For most of the brain regions (amygdaloid complex, hippocampus, posteroventral (inferior) parietal cortex, inferolateral temporal cortex, posterior (caudal) superior temporal cortex, anterior (rostral) cingulate (medial prefrontal) cortex, dorsolateral prefrontal cortex, ventrolateral prefrontal cortex, primary auditory cortex (core), orbital frontal cortex, primary visual cortex, and the primary somatosensory cortex), no genes passed our stringent criteria for elevated genes. The medial dorsal nucleus of the thalamus and the striatum were the exception, with 12 and 34 genes significantly elevated, respectively. No GO-terms were significantly enriched for the dorsal medial thalamus (12 genes). Elevated genes in the striatum were, among others, significantly enriched for dopaminergic synapse (*p* = 0.012) and dopaminergic synaptic transmission (*p* = 0.025), positive regulation of synaptic transmission, cholinergic (*p* = 0.021), and G-protein coupled receptor mediated signaling pathway (*p* = 5.48E^–8^) (Supplementary Figure 4). In conclusion, the enriched GO terms related to particularly high glutamate signaling were specific to the cerebellum. Thus, glutamate-related signaling might be a prime process conferring cerebellar vulnerability to ARCAs, including those caused by defective DNA repair pathways.

### 3.5. Disruption of glutamate signaling in the MB affects motor behavior, induces MB neuron DNA damage, and apoptosis

**FIGURE 5:**
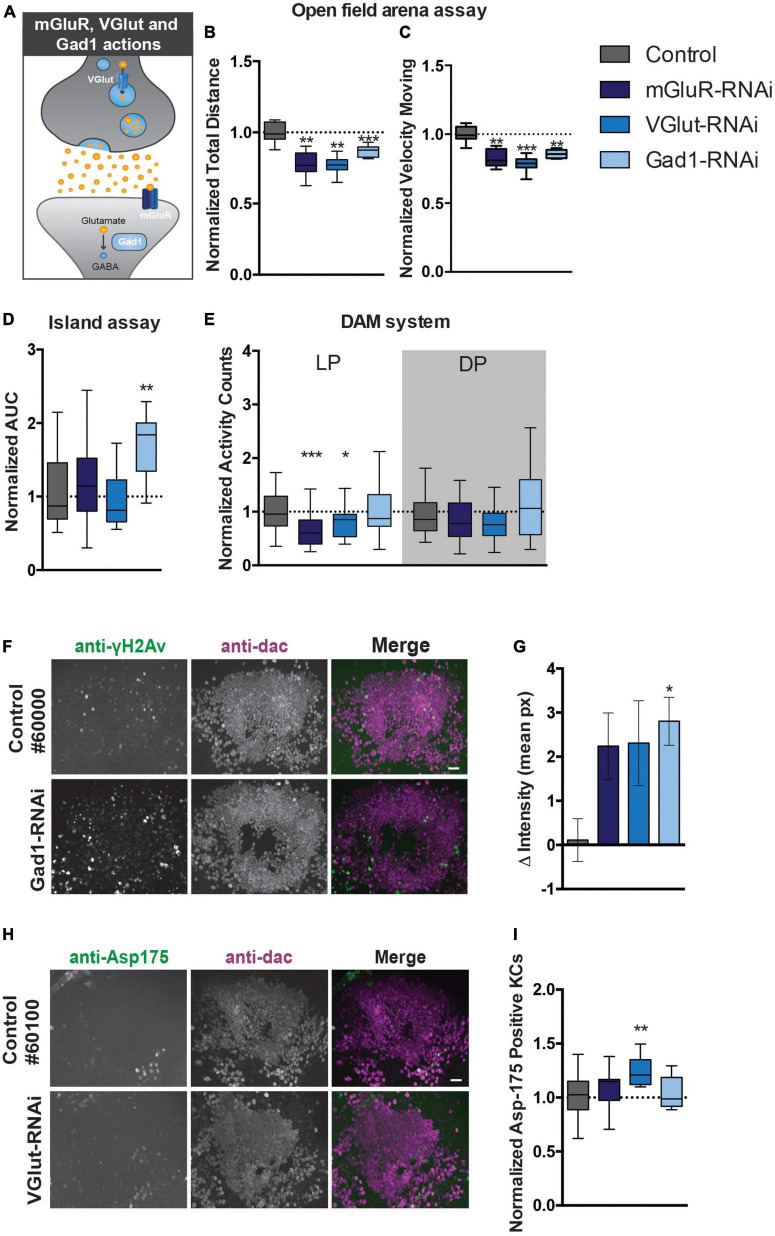
Perturbation of glutamate signaling in MB neurons affects motor behavior and causes DNA damage and apoptosis. **(A)** Scheme displaying the function of mGluR, VGlut, and Gad1 in the nervous system. **(B)**. Total distance moved and **(C)** speed while moving in the open field arena. Data is normalized against the appropriate genetic background control (RNAi/Control). **(D)** Area Under the Curve (AUC) for % of flies that remain on the platform during 10 s of island assay performance. Data is normalized against the appropriate genetic background control (RNAi/Control). **(E)** Normalized activity counts, measured with the DAM system. Data is normalized against control condition for Light Period (LP) and Dark Period (DP). (RNAi/Control) **(F)**. DNA damage and quantification: anti-γH2Av and anti-dac co-immunolabeling of brains of indicated genotypes. Scale bar: 10 μm, single optical sections are shown. **(G)** Quantification of DNA damage staining: bar-plots displaying mean γH2Av intensity in dac-positive Kenyon cell area–γH2Av intensity outside Kenyon cell area in brains of indicated genotypes. **(H)** Anti-Asp175 and anti-dac co-immunolabeling and **(I)** quantification. For information on genotypes and age, see Materials and methods. Scale bar: 10 μm, single optical sections are shown. Min-to-max boxplots displaying average number of dac-positive Kenyon cells that were positive for the cleaved-caspase-3 Asp175 marker for indicated genotypes. Data is normalized against appropriate control condition. ***p < 0.001, **p < 0.01, and *p < 0.05.

Cerebellar GCs are glutamatergic and previous studies indicated that developing KCs transiently express high levels of glutamate during the formation and maturation of the MB neural circuitry (Sinakevitch et al., 2010). We next started out to experimentally address to which extent motor behavior generated by the MB is dependent on glutamate signaling pathways. We therefore examined the effects of knocking down (i) the postsynaptic metabotropic glutamate receptor mGluR, (ii) the vesicular glutamate transporter (VGlut) that allows glutamate to enter synaptic vesicles, and (iii) the glutamic acid decarboxylase 1 (Gad1) that converts glutamic acid to the neurotransmitter GABA, on startle-induced and spontaneous motor behaviors (Figure 5A).

In the open field arena, mGluR-RNAi flies moved significantly less after startling (22% reduction, *p* < 0.01), and with significantly slower speed when moving (17% reduction, *p* < 0.01) (Figures 5B, C). Knockdown of mGluR with the MB 247-Gal4 driver did however, not affect island assay behavior (Figure 5D). MGluR knockdown affected spontaneous motor behavior. MGluR-RNAi flies passed the infrared beam in the DAM system significantly less often (33% reduction, *p* < 0.001) during the light period, compared to their genetic background control flies (Figure 5E). Thus, mGluR is involved in the regulation of startle-induced and spontaneous motor behaviors.

In the open-field arena, MB VGlut-RNAi flies moved significantly less (23% reduction, *p* < 0.01), and with slower speed (22% reduction, *p* < 0.001) (Figures 5B, C). Knockdown of VGlut in MBs did not affect island assay behavior (Figure 5D), but significantly affected spontaneous motor behavior in the DAM system. VGlut-RNAi flies showed a 20% reduction in activity counts in the light period (*p* < 0.05) compared to the control (Figure 5E). This suggests that VGlut, like mGluR, is important for startle-induced and spontaneous motor behavior.

Knockdown of Gad1 with the MB 247-Gal4 driver affected startle-induced, but not spontaneous motor behavior (Figures 5B–E). Like mGluR- and VGlut-RNAi flies, Gad1-RNAi flies walked a significantly shorter distance in a given time interval (reduction of around 20%, *p* < 0.001) compared to the controls, and, when moving, showed reduced velocity (reduction of around 20%, *p* < 0.01) in the open-field arena (Figures 5B, C). The AUC in the island assay of Gad1-RNAi flies showed a significant increase of 1.7-fold (*p* < 0.01) compared to their genetic controls (Figure 5D). Spontaneous motor behavior in the DAM system was unaffected (Figure 5E). Thus, Gad1 is important for startle-induced motor behavior.

In conclusion, perturbing glutamate signaling with the 247-driver by various manipulations appears to affect various aspects of motor behavior.

Having established that DNA repair and glutamate signaling are essential for aspects of motor function, we aimed to address whether both could be linked, hypothesizing that disruptive glutamatergic signaling leads to DNA damage and excitotoxicity in the MB. To experimentally test this hypothesis, brains of 247-Gal4 driven mGluR, VGlut and Gad1 knockdown flies were co-immunolabelled for γH2Av and dac, as described above. This revealed an increase in γH2Av intensity in the Kenyon cell area upon knockdown of all three genes, but was only confirmed to be significant after correction for multiple testing upon Gad1-RNAi (*p* < 0.05) (Figures 5F, G). We continued exploring whether the increase in γH2AV signal correlated with increased levels of active apoptosis. Knockdown of VGlut in the MBs led to a significant increase of Asp175 positive Kenyon cells (*p* < 0.01) (Figures 5H, I).

These results provide a first indication that affecting glutamate signaling in the MB can increase DNA damage and apoptosis, processes relevant to ARCAs resulting from mutations in ARCA-associated DNA repair genes.

### 3.6. Pharmacological reduction of glutamate signaling rescues the identified motor phenotypes

**FIGURE 6:**
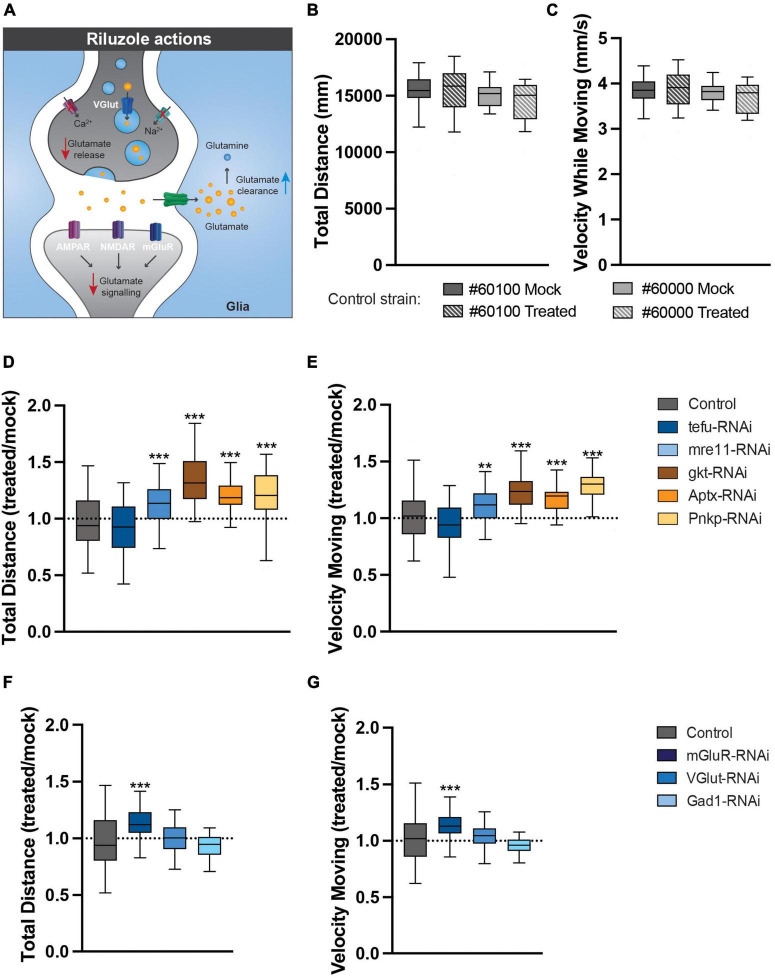
Pharmacological reduction of glutamate signaling rescues the identified motor phenotypes. **(A)** The actions of riluzole in the nervous system. **(B,C)** Riluzole treatment does not affect motor behavior of healthy controls. **(B)** Total distance (in mm) walked in open field arena for indicated control strains raised on Mock or 5 mM riluzole containing food. **(C)** Velocity while moving (in mm/s) in open field arena for indicated control strains raised on Mock or 5 mM riluzole containing food. **(D)** Total distance walked in open field arena for ARCA DNA repair genes is increased after 5 mM riluzole treatment. Plotted is total distance riluzole treated condition/total distance mock condition. **(E)** Velocity while moving in open field arena for ARCA DNA repair genes is rescued after 5 mM riluzole treatment. Plotted is velocity while moving riluzole treated condition/velocity while moving mock condition. **(F)** Total distance walked in open field arena for genes involved in glutamate signaling is rescued after 5 mM riluzole treatment. Plotted is total distance riluzole treated condition/total distance mock condition. **(G)** Velocity while moving in open field arena for genes involved in glutamate signaling is rescued after 5 mM riluzole treatment. Plotted is velocity while moving riluzole treated condition/velocity while moving mock condition. ***p < 0.001, **p < 0.05. For information on age and genotypes, see section “2. Materials and methods”.

Finally, to address whether glutamate excitotoxicity contributes to the motor phenotypes of our ARCA DNA repair and glutamate signaling *Drosophila* models, freshly eclosed flies were transferred to and raised on either mock food, or food containing 5 mM of the glutamate signaling antagonist riluzole (Figure 6A). Riluzole inhibits the release of glutamate, stimulates the reuptake from glutamate in the synaptic cleft by glia and has been shown to be linked to reduced activity of voltage-gated Ca^2+^ and Na^2+^ channels (Figure 6A; Blyufer et al., 2021). At day 4, spontaneous, startle-induced motor behavior was evaluated in the open field arena. At the utilized concentration, riluzole treatment did not affect motor behavior of healthy control strains crossed with the 247-Gal4 driver in the open field arena (Figures 6B, C). Interestingly though, it significantly improved motor behavior in our ARCA DNA repair and glutamate signaling models (Figures 6D–G). Mre11 (12.1% improvement, *p* < 0.01) Gkt (34% improvement, *p* < 0.001), Aptx (21.7% improvement, *p* < 0.001), Pnkp (20.5% improvement, *p* < 0.001), and mGluR (13.0% improvement, *p* < 0.001) RNAi flies moved significantly more compared to their Mock-treated siblings (Figures 6D, F). In these models, a significant improvement in the velocity while moving was observed as well after riluzole treatment. Mre11 (10.7% improvement, *p* < 0.01) Gkt (23.5% improvement, *p* < 0.001), Aptx (15.0% improvement, *p* < 0.001), Pnkp (26.9% improvement, *p* < 0.001), and mGluR (13.0% improvement, *p* < 0.001) RNAi flies moved significantly faster compared to their Mock-treated siblings (Figures 6D, F). No difference was found upon treatment of tefu, VGlut and Gad1 RNAi flies under the utilized experimental conditions.

In conclusion, glutamate excitotoxicity convers vulnerability of motor circuits upon loss of ARCA DNA repair and glutamate signaling genes.

## 4. Discussion

In this study, we investigated the relation of the *Drosophila* MB – a structure in the fly brain reported to have structural similarities to the cerebellum (Farris, 2011) – to motor behaviors. We report that disruption of the MB affects efficient startle-induced and spontaneous locomotor behaviors. We further propose that the functionality of the MB in motor behavior depends on ARCA-associated DNA repair and on proper dose of glutamate signaling, and that glutamate excitotoxicity confers cerebellar vulnerability in ARCA-associated DNA repair disorders.

### 4.1. The *Drosophila* MB, a cerebellar-like structure involved in motor behavior?

The here described role of the MB in startle-induced and spontaneous motor behavior is supported by several independent approaches and is in line with previous publications demonstrating a role for the MB in startle-induced climbing behavior, flight behavior and gap climbing behavior (Arena et al., 2017; Sun et al., 2018; Manjila et al., 2019). Our work complements these findings and demonstrates for the first time that MB neurons also plays a role in island assay behavior and walking behavior in an open-field arena. The conducted Split-Gal4 experiments suggest that all tested MB cellular subsets contribute to these behaviors. In contrast, whether motor defects arise from developmental consequences of inhibited synaptic transmission in MB neurons or acute functions of specific MB neuronal cell types in motor behavior remains to be addressed.

In the cerebellar circuit, visual and sensory information is constantly integrated to predict, generate and update coordinated motor behavior. The expansion layer of the cerebellum makes it uniquely suited for this. Individuals with cerebellar ataxia show specific deficits in somatosensory perception during active movements (Peterburs and Desmond, 2016; Therrien and Bastian, 2019). This suggest that ataxic movements are consistent with disrupted prediction of motor movements rather than impaired primary sensory or motor function. Like the cerebellum, the MB is an integration center. Environmental information, such as visual and olfactory cues, is conveyed to and represented in the MB, which in turn can be used to modulate coordinated motor output behavior (Lewis et al., 2015; Arena et al., 2017). For example, in gap-climbing tasks, flies visually evaluate the gap width and modulate motor programs to climb over the gap. Upon repeated exposure to gaps of similar widths, the fly can improve its climbing ability, which represents a form of motor skill learning that depends on the MB. It is interesting to speculate that the motor defects observed in this study are due a reduced representation of sensory stimuli by available, healthy Kenyon cells in the MB expansion layer, which in turn affects sensory integration and the generation of coordinated motor output. This remains to be further investigated.

### 4.2. Function of ARCA-associated DNA repair pathways in the MB

We demonstrated that knockdown of ARCA-associated DNA repair genes induced by the 247-Gal4 driver, strongly expressed in α/β-lobes and γ-lobes of the MB (Kaun et al., 2011; Pech et al., 2013), affects startle-induced and spontaneous motor behaviors. This suggests that DNA repair is needed to develop and/or maintain the integrity of MB neurons and their output motor programs. How could functional ARCA DNA repair pathways contribute to cerebellar-like circuit function? DNA damage continuously takes place in living cells and can arise from endogenous factors, such as reactive oxygen species (ROS), but also occurs as a byproduct, e.g., during the division of cerebellar granule cell progenitors (Caldecott, 2008; Andres et al., 2015). The post-mitotic nature and relatively long lifespan of neuronal cells might make them particular sensitive to defects in DNA damage pathways. While this applies to most neurons, the striking cerebellar vulnerability in ARCA DNA repair disorders suggest that DNA repair is a key homeostatic process in cerebellar-like circuits. This might be due to their unique expansion layer, the enormous number of excitatory GCs predisposing to glutamate excitotoxicity, the high metabolic activity, the high oxidative load, and intrinsic firing properties of neurons that make up the cerebellum (Madabhushi et al., 2014; Synofzik et al., 2019). In addition, cerebellar granule cells, being the most abundant cell types in the brain, proliferate during development by massive expansion. This could generate replication stress-associated DNA damage, affecting the developing granule (-like) cells (such as KCs)–and indirectly other cerebellar-like cell types to which they signal or receive information from. The early (day 4) behavioral phenotypes upon knockdown of ARCA-associated DNA repair genes support a developmental component of ARCA-associated DNA repair pathways in the MB. This is in agreement with a previous study demonstrating a role for one of the investigated genes, gkt, in axonal bifurcation of MB neurons (Nitta and Sugie, 2017). Interestingly, the expression of ARCA-associated DNA repair genes is higher in the prenatal than postnatal cerebellum, also hinting at an important developmental role of ARCA DNA repair genes during the establishment of the cerebellar circuitry (Eidhof et al., 2018b). It is therefore particular exciting that a rescue of the ARCA-associated motor phenotypes was achievable by adult administration of the glutamate release inhibitor riluzole. This finding might bear therapeutic potential.

In our study, knockdown of ARCA-associated DNA repair genes in the MB did not consistently lead to the same DNA damage and/or apoptotic and morphology phenotypes. Regardless, in terms of motor behavior, knockdown of ARCA-associated DNA repair genes led to highly consistent phenotypes. Inconsistencies on the cellular level might be explained by differences in RNAi knockdown efficiency, differences in critical expression thresholds of the respective proteins related to their functions, RNAi off-targets, differences in expression patterns of the ARCA DNA repair genes (Supplementary Figure 5) and the stage of pathology we looked at. Since the cellular markers that were used only detect damage at the monitored developmental age (day 20 after eclosion), it is possible that the relevant time-point for active damage (e.g., ongoing cell-death) was missed for some genotypes. To explore whether we potentially missed active apoptosis at an earlier stage in the ARCA DNA repair models, we performed quantifications of the number of Kenyon cells present in our Day 20 stainings, for one genotype that showed a significant increase in cleaved caspase-3 at day 20 (mre11) and for one that we did not (Aptx) (Supplementary Figure 6). The results mirror the results of our cleaved caspase-3 stainings in these mutant conditions at day 20 (Figure 3D): whereas Mre11 was characterized, as expected after the detected cell death, by a significant decrease in Kenyon cell number, the number in Aptx was unaffected. We therefore conclude, that although apoptosis may start at an earlier age, it is unlikely that undetected cell death at that stage plays a systematic role in the differences we observed. Another, more likely explanation is the divergent functions that ARCA DNA repair genes serve. Indeed, dependent on the DNA lesions that arise in response to damaging conditions, the investigated ARCA DNA repair genes can function in partially overlapping, but also different DNA repair pathways [(Synofzik et al., 2019; Table 1)]. Tefu is a protein kinase that phosphorylates cell-cycle and DNA repair proteins to inhibit the cell cycle and activate DSB DNA repair upon DSB damage. It is likely that upon Tefu knockdown, signaling pathways to repair DSBs are no longer intact, resulting in increased DNA damage during the clonal expansion and differentiation of Kenyon cells. Since Tefu-mediated phosphorylation is also important for pro-apoptotic signaling, this process is likely impaired as well and dysfunctional Kenyon cells might persist in the brain and contribute to the observed behavioral phenotypes. Mre11 has endonuclease and 3′ to 5′ exoncuclease activity which is required for non-homologous joining of DNA ends during DSB repair (Rulten and Caldecott, 2013). Loss of Mre11 might thus result in dysfunctional DSB repair. It is conceivable that this process is important during the clonal expansion of Kenyon cells, but also for their maintenance (e.g., to repair DSBs caused by glutamate receptor activation and excitotoxicity). The orthologs of gkt, Aptx and Pnkp have all been described to catalyze DNA strand break termini and are involved in single-stranded DNA break repair (SSBR). The ortholog of gkt processes topoisomerase 1 (TOP1)-DNA phosphotyrosyl bonds to form gkt-DNA intermediates, which subsequently undergo gkt-mediated hydrolysis (Jiang et al., 2017). Aptx catalyzes the removal of 5′-adenosine monophosphate (AMP) from abortive DNA ligation intermediates, whereas Pnkp phosphorylates 5′-OH termini and dephosphorylates 3′-phosphate termini (Jiang et al., 2017). SSBR appears highly relevant for the maintenance of Kenyon cell DNA as SSBs are a frequent consequence of glutamate excitotoxicity induced stress (Konopka and Atkin, 2022). In contrast, while DSBs occur less frequent upon glutamate excitotoxicity induced stress compared to SSBs, these are overall more damaging to neurons (Konopka and Atkin, 2022). The expression of glutamate receptors are also linked to DSBs. These DSBs resolve topological limitations to neural gene expression by use of for example NHEJ (Konopka and Atkin, 2022). DNA damage and repair may thus alter the expression and activity of glutamate receptors as well, thereby affecting neural transmission. Thus, dependent on the DNA lesions that arise in response to different damaging conditions, the investigated DNA repair genes can function in partially overlapping, but also different DNA repair pathways that may be crucial at different developmental times for different processes and may thus vary in different neurons and circuits. It is therefore conceivable that phenotypes upon knockdown of different ARCA-associated DNA repair genes in the MB overlap, but can also differ between genes to certain extents. In agreement with this, cerebellar pathologies do not necessarily overlap between ARCA-associated DNA repair genes (Rulten and Caldecott, 2013; Synofzik et al., 2019). Moreover, phosphorylation of H2Av, which is a biomarker of Double Stranded Breaks (DSBs) and sites of DNA repair foci, serves as one of the early events in the DNA damage response. Upon DSBs, ATM, the ortholog of tefu, is activated by the MRE11A-RAD50-NBS1 (MRN) complex and phosphorylates H2Av. γH2Av in turn allows recruitment of the MRN complex to DSB foci to initiate DNA repair. It is very likely that we do not observe a significant increase in γH2Av signal in MB Kenyon cells upon knockdown of mre11 and tefu, because both proteins function upstream of γH2Av. The function of Pnkp in NHEJ DNA repair is downstream of γH2Av, which explains the specific increase in γH2Av intensity in the Kenyon cell area upon Pnkp knockdown with the 247-Gal4 driver. Importantly, MRE11A-, APTX- and TDP1-induced DNA repair has also been shown to be largely independent of γH2Av (Mirzoeva and Petrini, 2003; El-Khamisy et al., 2009). Examination of other DNA break and cell stress markers would contribute further to understand the function of all ARCA-associated DNA repair genes in the MB and early stages of MB pathology.

### 4.3. The role of glutamate signaling in ARCA DNA repair disorders, cerebellar, and MB function

Our analysis revealed that genes involved in glutamate signaling are expressed at much higher level in the human cerebellum compared to the rest of the brain, across postnatal developmental stages. This likely is explained by the enormous number of glutamatergic granule cells, that can receive glutamatergic input from mossy fibers (MF), and provide excitatory glutamatergic output to cerebellar Purkinje cells. The MF-GC and GC-PC interaction can thus make the cerebellar circuitry particularly vulnerable to disruptions in glutamate signaling. Glutamate receptors, such as GRM1, are highly expressed in the cerebellar circuit (Kano and Watanabe, 2017). Interestingly, both gain and loss-of-function mutations in *GRM1* have been described in cerebellar ataxias (Watson et al., 2017; Bossi et al., 2018). This suggests that mutations in genes involved in glutamate signaling that are highly upregulated in the cerebellum can cause ataxia, either via excess activation or loss of glutamate signaling. New born Kenyon cells that make up the MB also express high levels of glutamate (Sinakevitch et al., 2010). *Drosophila* only has one metabotropic glutamate receptor, mGluR. Knockdown of mGluR using the 247-Gal4 driver affected startle-induced motor behavior, suggesting a conserved role for metabotropic glutamate receptors in the MB and motor behavior. The vesicular glutamate transporter VGLUT1 is highly expressed in the granule cell layer of the human cerebellar cortex and reduced VGLUT1 expression has been reported in several cerebellar ataxia animal models (Tempia et al., 2015; Vigneault et al., 2015; Lin et al., 2017). Knockdown of the VGLUT1 *Drosophila* ortholog VGlut by use of the 247-Gal4 driver significantly affected startle-induced and spontaneous motor behavior and led to a significant increase in apoptotic Kenyon cells. This suggests a potential conserved function for VGlut in the MB, a cerebellar-like structure. While reduced glutamate signaling is corrupting locomotor function in flies and some patients with ataxia, another possible wider theme in ataxia might be excitotoxicity evoked by high levels of glutamate. Release of glutamate in the synaptic cleft induces postsynaptic calcium influx. Intracellular calcium levels are directly proportional to mitochondrial activity and can lead to ROS overproduction, increased levels of DNA damage and ultimately neuronal death (Yang et al., 2011). Interestingly, we were able to demonstrate that pharmacological reduction of glutamate signaling can rescue the motor phenotypes of our ARCA DNA repair models, suggesting that excitotoxicity indeed plays a role and confers vulnerability in ARCA DNA repair disorders. Moreover, mGluR has been associated with excitotoxicity due to disrupted feedback mechanisms between pre- and post-synapses (Xu et al., 2007; Crupi et al., 2019). In agreement with this, pharmacological reduction of glutamate signaling rescued the motor phenotypes of our mGluR *Drosophila* model in the open-field arena.

Another approach we took to increase intracellular glutamate levels in the MB, was to knock down the enzyme glutamate decarboxylase (Gad1) that processes glutamic acid to GABA. Gad1 loss-of-function in *Drosophila* has previously been linked to increased levels of glutamate and excitotoxicity (Romano et al., 2018). In humans, autoantibodies against GAD1 have been associated with cerebellar ataxia (Mitoma et al., 2016). Gad1 loss-of-function in the *Drosophila* MB led to motor defects and a significant increase in γH2Av signal in Kenyon cells, suggestive of DSBs. Notably, Pnkp knockdown with the 247-Gal4 driver induced the same increase in γH2Av intensity in Kenyon cells, implying that functional DNA repair pathways might be required to prevent detrimental consequences upon loss of Gad1. Nevertheless, we were not able to rescue the motor phenotypes of Gad1 in the open-field arena by pharmacological reduction of glutamatergic signaling. At this moment it remains thus unclear to which extent the behavioral phenotypes upon Gad1 knockdown are due to increased levels of DNA damage and glutamate excitotoxicity. Gad1 knockdown also leads to a reduction of the inhibitory neurotransmitter GABA, and at present we do not know to which extent this contributed to the observed locomotor phenotypes and increased γH2Av signal in Kenyon cells. Nevertheless, our different genetic approaches, including the riluzole rescue experiments lead us to suggest that alterations in glutamate signaling is implicated in the observed ARCA-DNA repair locomotor phenotypes and that the adjustment of this dysregulation can help to prevent them.

In conclusion, the findings here highlight the importance of ARCA-associated DNA repair genes and glutamate signaling pathways to the human cerebellum, the *Drosophila* MB and locomotor behavior. Since the interplay between cerebellar vulnerability, glutamate signaling and functional DNA repair pathways can very much depend on the specific DNA repair gene that is mutated, and the specific characteristics of neuronal cell types in the cerebellum, more research is required to understand how these mechanisms ultimately contribute to the emergence of cerebellar ataxia. It would be interesting to explore further whether targeting glutamate signaling in ARCA DNA repair disorders can also alleviate DNA damage, apoptosis and other startle-induced and spontaneous motor behaviors, such as those identified in the island assay and with the DAM system. If this is indeed the case, it appears valuable to further test the therapeutic potential of glutamate intervention in mammalian models of ARCA-associated DNA repair genes.

## Data availability statement

The original contributions presented in this study are included in the article/Supplementary material, further inquiries can be directed to the corresponding authors.

## Ethics statement

Ethical review and approval was not required for the animal study. Arthropods used in this study do not require approval by an animal Ethics Committee.

## Author contributions

IE, BW, and AS conceived the study and interpreted the results, wrote and critically reviewed the manuscript. IE and AK designed the analyses, collected, analyzed, and interpreted the data. All authors read and approved the final version of the manuscript.

## Footnotes

1
http://www.brainspan.org
