# Supplementary material for: Ataxia-associated DNA repair genes protect the Drosophila mushroom body and locomotor function against glutamate signaling-associated damage
Source: Front Neural Circuits. 2023 Jul 5;17:1148947. doi: 10.3389/fncir.2023.1148947 (PMC10354283; doi:10.3389/fncir.2023.1148947)
Supplement: Supplementary file 1 [file Data_Sheet_1.pdf]

Supplementary Material

Ataxia-associated DNA repair genes protect the *Drosophila* mushroom body and locomotor function against glutamate signaling-associated damage

Ilse Eidhof[1,2], Alina Krebbers[1,3], Bart van de Warrenburg[4] and Annette Schenck[1]

[1] Department of Human Genetics, Donders Institute for Brain, Cognition and Behavior, Radboud University Medical Center, Nijmegen, The Netherlands.

[2] current address: Department of Medical Biochemistry and Biophysics, Karolinska Institute, Stockholm, Sweden.

[3] current address: Department of Physiology, Anatomy and Genetics, University of Oxford, Oxford, The United Kingdom.

[4] Department of Neurology, Donders Institute for Brain, Cognition, and Behaviour, Radboud university medical center, Nijmegen, The Netherlands.

*** Correspondences to:**
Dr. Ilse Eidhof
Ilse.Eidhof@ki.se

Prof. Annette Schenck
Annette.Schenck@radboudumc.nl

Supplementary Figures

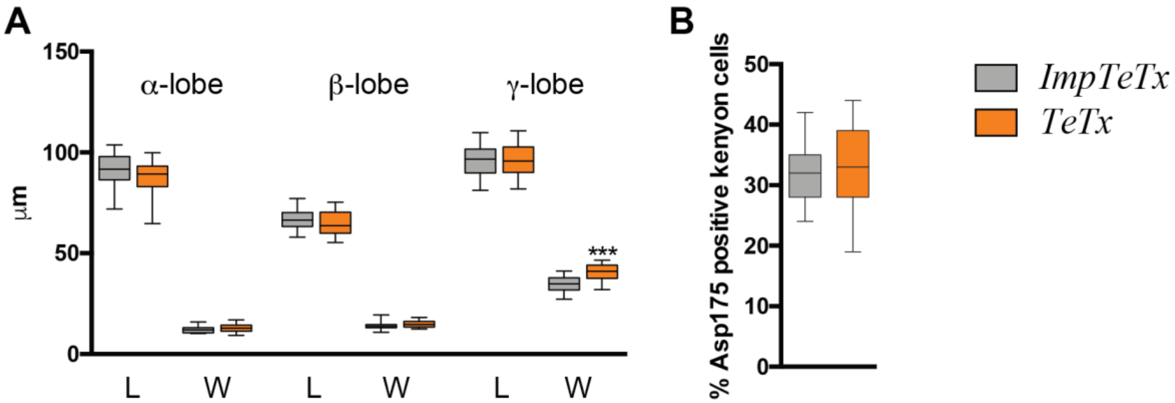

Supplementary figure 1: Inhibition of synaptic transmission does neither induce gross structural changes to mushroom body morphology, nor induces apoptosis of Kenyon cells. A. Min-to-max boxplots displaying average length and width (in µm) of indicated MB lobes and genotypes. MB lobes were visualized using an antibody against Fasciclin-II. L: Length, W: Width. *** $p < 0.001$. **B.** Min-to-max boxplots displaying average number of Kenyon cells that were positive for the cleaved-caspase-

3 Asp175 marker. Age of flies: 20 d. Genotypes are ImpTeTx: *;UAS-impTeTx/+;R13F02-Gal4/+* (control expressing impaired Tetanus toxin) and TeTx: *;UAS-TeTx/+;R13F02-Gal4/+* (synaptic transmission inhibited by expression of Tetanus toxin).

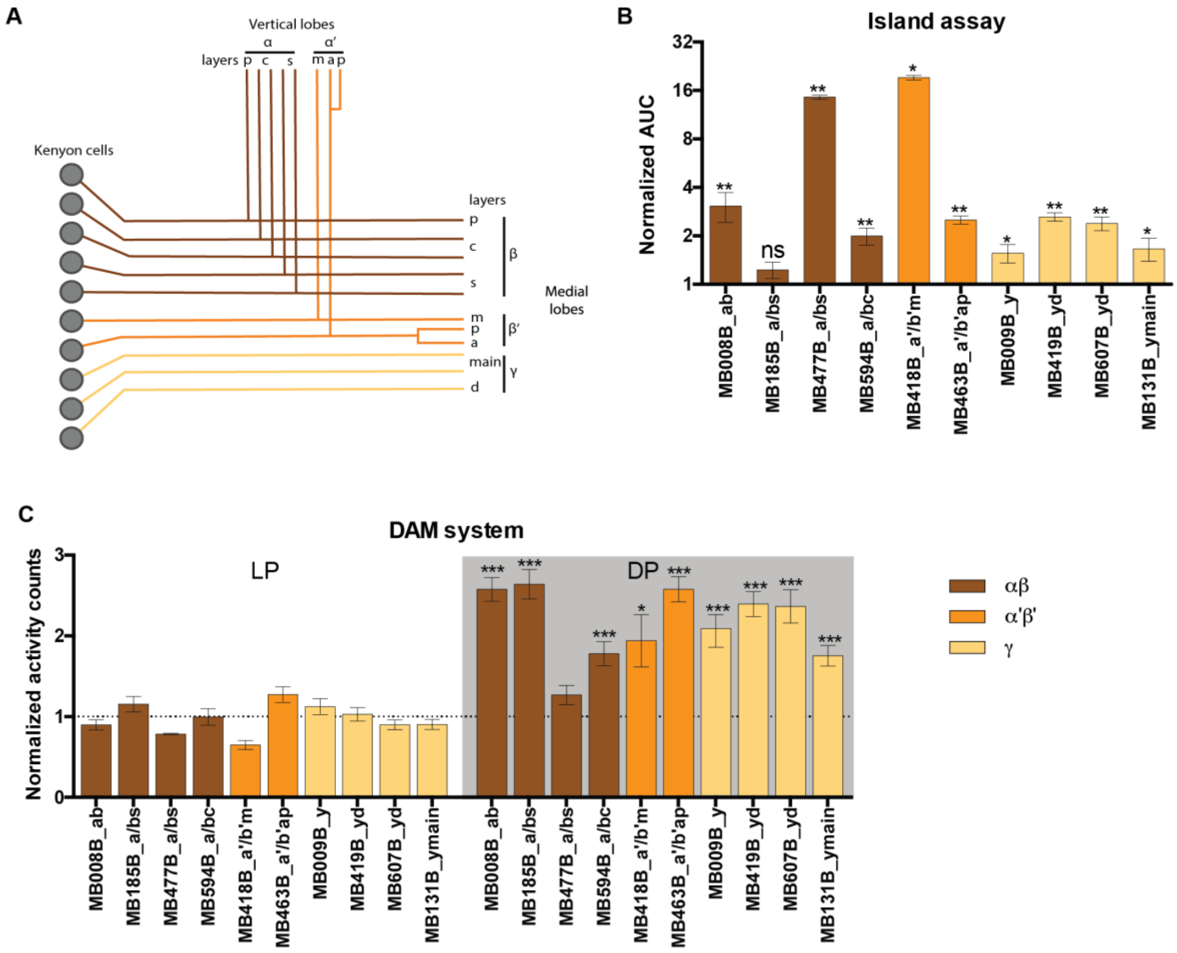

Supplementary figure 2: Inhibition of synaptic transmission by targeted expression of TeTx in subsets of mushroom body neurons affects motor behavior. A. Schematic representation of neuronal wiring in the MB. **B.** Area Under the Curve (AUC) for % of flies that remained on the island assay platform. Data is normalized against the appropriate genetic background control (MB Split-Gal4 driver crossed to TeTx/MB Split-Gal4 driver crossed to impTeTx); a value of 1 indicates no changes compared to control condition. **C.** Activity counts, measured with the DAM system. Light Period (LP) and Dark Period (DP). Each value is normalized against its appropriate control (TeTx/ImpTeTx) in the respective period, MB Split-Gal4 driver and period as indicated; a value of 1 (dotted line) indicates no changes compared to the control. Age of flies: 4 d. For information on genotypes, see Materials and methods.

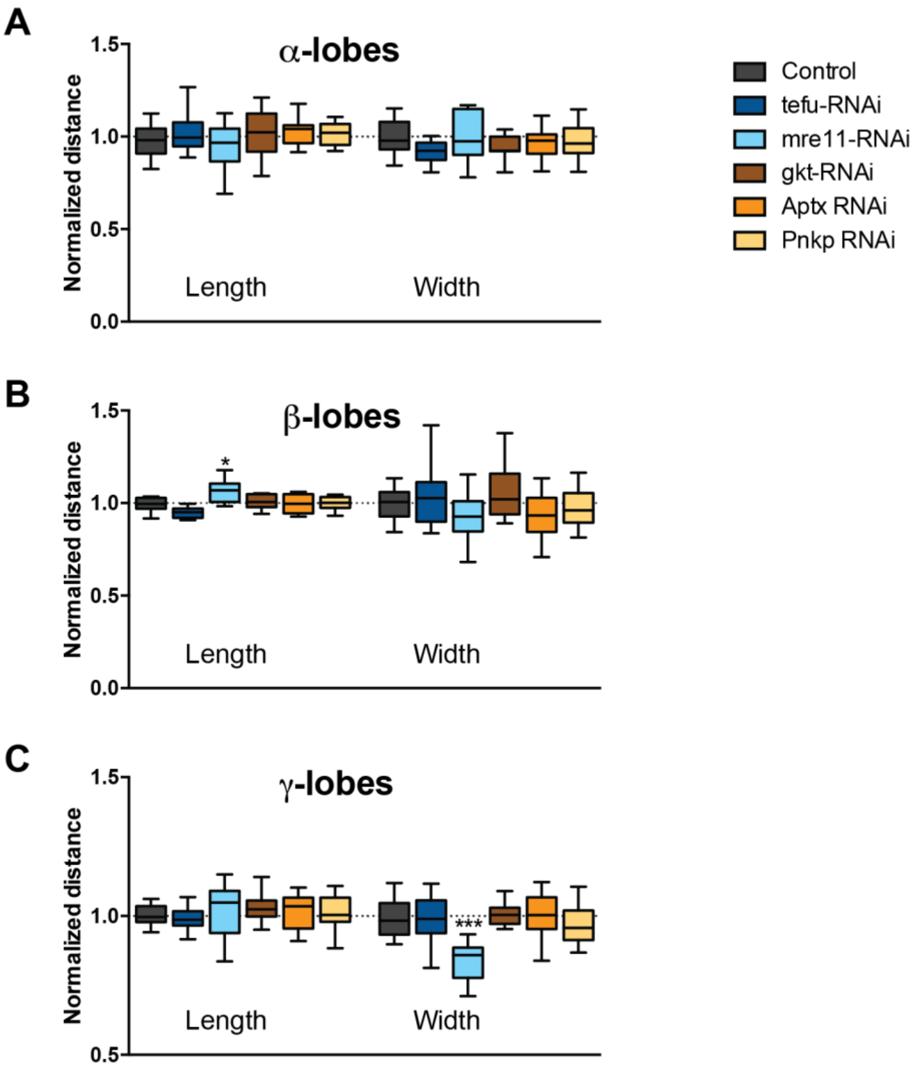

Supplementary figure 3: Loss of ARCA-associated DNA repair genes in the MB does not induce gross structural changes in MB morphology. A-C. Min-to-max boxplots displaying average length and width (in µm) of indicated MB lobes and genotypes. MB lobes were visualized using an antibody against Fasciclin-II. * $p < 0.01$, *** $p < 0.001$. Age of flies: 20 d. For information on genotypes, see Materials and methods section.

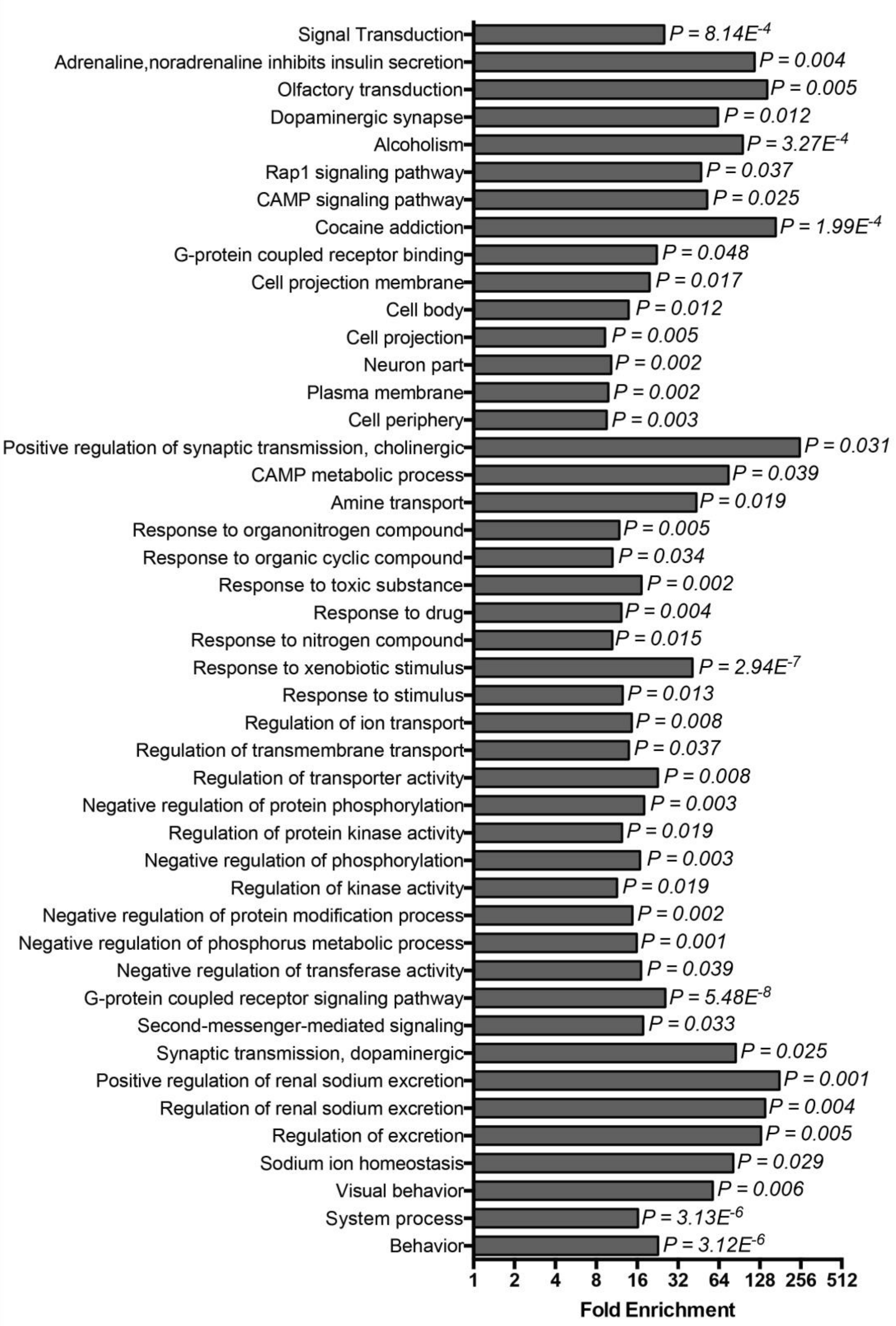

Supplementary figure 4: Gene Ontology terms enriched for genes highly, specifically, and persistently expressed (>10-Log2fold enrichment, *p-adj*. <0.05) in the postnatal striatum.

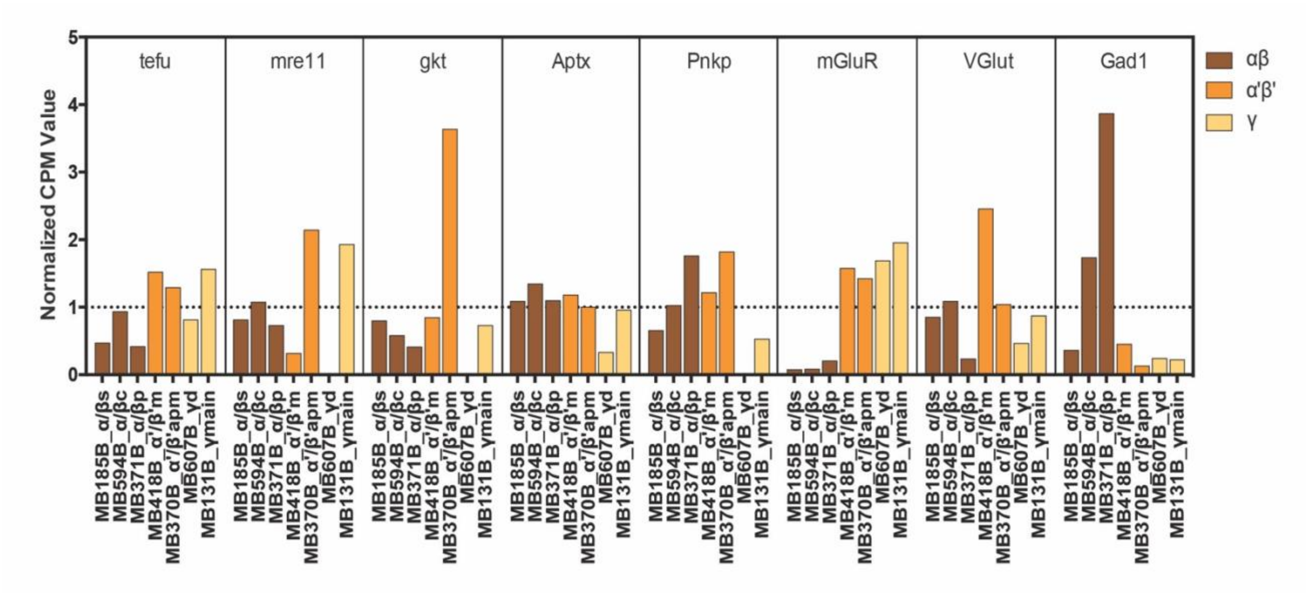

Supplementary figure 5: Expression data of isolated nuclei of specific MB neuronal subtypes for all genes investigated in this study. Data are from Shih et al.[25]. Per gene, expression values in different MB neuronal subtypes were normalized against the average Counts Per Million (CPM) value of that specific gene over all MB neuronal subtypes. Values <1 indicate that the expression in the specific MB neuronal subtype was lower than the average expression of the specific gene in the MB, values >1 indicate higher expression. X-axis displays the Split-Gal4 lines from which the nuclei were isolated.

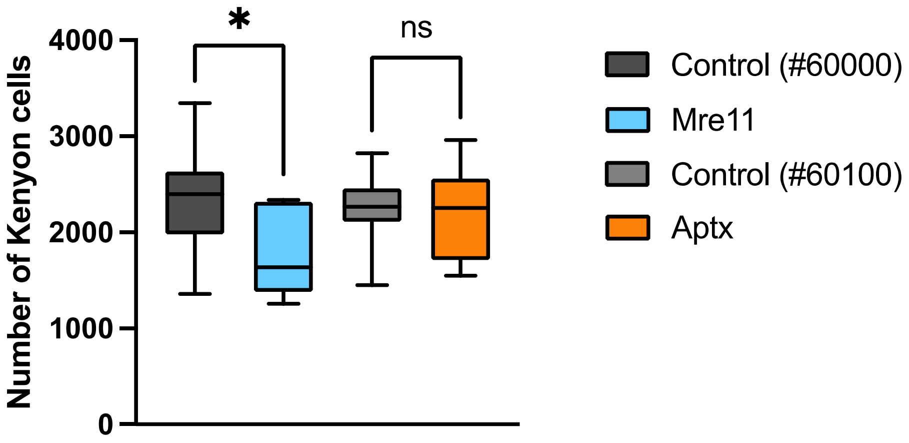

Supplementary figure 6: Quantification of the number of Kenyon cells per hemisphere for the indicated genotypes crossed to the 247-Gal4 driver at day 20.
